# Associations Between the Use of Fitness and Diet Tracking Technology and Disordered Eating Behaviour: A Systematic Review

**DOI:** 10.1002/erv.70006

**Published:** 2025-07-10

**Authors:** Sarah Moody, Lindsay Ross, Marie‐Christine Opitz, Amelia Hemmings, Başak İnce, Callum Bryson, Carina Kuehne, Daire Douglas, Matthew Phillips, Vivienne Langhorne, Ulrike Schmidt, Helen Sharpe

**Affiliations:** ^1^ School of Heath in Social Science University of Edinburgh Edinburgh UK; ^2^ Centre for Research in Eating and Weight Disorders Department of Psychological Medicine Institute of Psychiatry, Psychology and Neuroscience King's College London London UK; ^3^ South London and Maudsley NHS Foundation Trust London UK

## Abstract

**Background:**

The fitness and wellbeing technology industry is growing rapidly. Concerns are emerging regarding whether these may increase disordered eating behaviours. This review is the first to systematically examine the relationship between fitness and diet tracker use and disordered eating in general and clinical populations.

**Methods:**

The following databases were searched: EMBASE, Medline/PubMed, PsychInfo, CINAHL Plus, ERIC, SportDiscus, ASSIA, Social Science Premium, Sociological Abstracts, Sports Medicine and Educational Health, SCOPUS, Cochrane Library, and ProQuest Dissertation and Theses Global. Studies were selected using predetermined inclusion and exclusion criteria. A narrative synthesis was used, and results were reported by disordered eating outcome.

**Results:**

Twenty‐seven studies were included in the final review. Cross‐sectional studies revealed reasonably consistent evidence of an association between disordered eating and fitness and diet tracker use, specifically regarding global disordered eating, dietary restraint, excessive exercise, and disordered muscle‐orientated behaviour. However, this association was not replicated in experimental research.

**Conclusion:**

While fitness and diet tracker use is a correlate of disordered eating, it is currently not possible to conclude if they increase disordered eating, or the direction of this relationship. Future research should determine the nature of this relationship and possible mechanisms to ensure their safe use in vulnerable populations.

## Summary

- Fitness and diet trackers (FDTs) are common technologies designed to improve health
- Cross‐sectional and observational studies, but not experimental studies revealed an association between FDTs and disordered eating.
- More research is needed on FDT use, especially in vulnerable groups

## 1 Introduction

The past 15 years have seen exponential growth in the fitness and well‐being device industry (Statista Research) These technologies include phone applications (apps) for tracking dietary intake and exercise, as well as wearable devices with inbuilt sensors to record real‐time activity and physiological markers. Fitness and diet trackers (hereafter referred to as FDTs) are designed to promote healthy lifestyles by allowing users to set goals, track progress, and ‘compete’ with others (Sullivan and Lachman 2017). Research has previously demonstrated beneficial effects of health behaviour change techniques such as goal‐setting and self‐monitoring in reducing sedentary behaviour and promoting healthy eating (Samdal et al. 2017; Compernolle et al. 2019). It is therefore feasible that FDT use may be associated with positive health outcomes via supporting these behaviour change strategies.

That said, critical discourse has emerged on possible negative consequences associated with FDT use. Primarily, concerns have been raised that FDTs may increase the risk for disordered eating and/or eating disorders (EDs) via promoting dieting behaviours and preoccupation around weight, shape and eating (Honary et al. 2019). EDs, such as anorexia nervosa and bulimia nervosa, are highly disabling mental disorders (Treasure et al. 2020) which can lead to poor mental and physical health outcomes (Micali et al. 2015). Disordered eating can include a variety of behaviours and cognitions, including dietary restraint, the cognitive effort to limit food intake (Stewart et al. 2022), persistent overvaluation of an individual's weight and/or shape (Murphy 2017), binge eating episodes (American Psychiatric 2013) and compensatory behaviours such as vomiting and the use of laxatives (Abebe et al. 2012). Additionally, compulsive exercise, which includes excessive exercise, is a possible symptom of EDs, in which an individual follows strict rules regarding exercise in a rigid manner with a goal of reducing distress or preventing negative consequences (Dittmer et al. 2018). Whilst disordered eating behaviours are not necessarily indicative of an ED, they are associated with negative outcomes and can be risk factors for later, more clinically significant, difficulties (Stice et al. 2021).

Design features of FDTs, including goal setting, the ‘gamification’ of tracking, and reminders/notifications, may exacerbate the risk of developing EDs by promoting high‐intensity and prolonged use of FDTs (Honary et al. 2019). These concerns are mirrored by the lived experience of people using FDTs, some of whom have reported developing obsessions with the ‘numbers’ (e.g., calorie counts, step counts), developing rigid rules around eating and activity, and setting increasingly extreme goals (Eikey 2021). Whilst some FDTs have implemented features aiming to minimize risky behaviours (e.g., notifications that caloric intake is very low), evidence suggests that people commonly find ways around these measures (McCaig et al. 2020), or find them to be motivating rather than discouraging (Eikey 2021).

Specific concerns have been raised regarding the use of FDTs by people with EDs. Eikey and Reddy (2017) conducted interviews with 16 individuals with an ED who used the app MyFitnessPal and reported 59% of the sample felt ‘obsessed’ and ‘compelled’ to log their calories. Participants also described feeling the need to restrict their diet to reach the app's goals and engaged in purging behaviours if they exceeded their limits. Mentally tracking caloric intake is already a common behaviour of EDs (Lavis 2018). As such it is possible that FDTs may make this method more accessible to those at risk of an ED or reduce the mental taxation required and promote this behaviour in those already tracking their intake. This element of ‘gamification’ alongside features promoting continued use, may make FDTs potentially harmful for specific vulnerable populations. It is therefore important to understand to what extent FDT use may exacerbate or maintain disordered eating behaviours in these clinical groups.

In addition to those with EDs, it is feasible that there are other groups who may be more vulnerable to developing disordered eating behaviour through FDT use. For example, it may be that body size moderates risk in this context, with those in larger bodies potentially being at heightened risk given the greater sociocultural pressures around appearance in these groups (Eisenberg et al. 2006; Olson et al. 2021). Increasingly FDTs are being used as part of weight management programmes, so those in larger bodies may be more likely to use these devices with motivations related to changing body weight and shape (rather than, e.g., increasing fitness, or improving nutritional intake) (Cheatham et al. 2018). Additionally, due to the ubiquity of technology use within younger generations (Wartella et al. 2016), alongside the increase in prevalence rates of EDs during adolescence (Schmidt et al. 2016), the impact of FDT use could vary by age group. Being able to identify potentially vulnerable groups will be important for targeting preventative interventions effectively.

As FDTs are becoming increasingly common, understanding the impact of this technology is a vital precursor to being able to provide guidance regarding appropriate use of these tools. Existing systematic reviews have shown that wearable activity trackers are associated with increases in physical activity, particularly higher step counts (Ferguson et al. 2022). Evidence for the impact on other health outcomes, however, is less clear (Ferguson et al. 2022). Roth et al. (2024), have explored the association between weight‐related self‐monitoring (including FDT use) in the general population and report no association between this and disordered eating symptoms, although they highlight that looking at the general population as a whole may mask associations in high‐risk groups. Other reviews have considered how recovery apps are used by those with an eating disorder, highlighting that some of these include diet tracking features (Wasil et al. 2021). Building on these existing reviews, the current review aims to answer the following research questions:

1. Is there an association between the use of FDTs and disordered eating?
2. Are there any vulnerable groups who are at greater risk of negative outcomes when using FDTs?

## 2 Methods

The review protocol was registered on the Open Science Framework (https://doi.org/10.17605/OSF.IO/DRVNY) and written up in line with the Preferred Reporting Items for Systematic Reviews and Meta‐Analyses (PRISMA) guidelines (Page et al. 2021).

### 2.1 Eligibility Criteria

Articles were eligible if they met the following criteria:

1. Included a measure of fitness and/or diet tracking technology use. This includes a smartphone app, such as MyFitnessPal, and/or a heart rate monitoring wearable device, such as a Fitbit.
2. Included a measure of disordered eating symptomology. This includes an ED diagnosis or measure of disordered eating such as excessive exercise, food restrictions, fasting, purging, or binging behaviour.
3. Were published from 2005 onwards. This reflects the period where fitness tracking technology came into common use in the general population. (Zheng et al. 2018; Breslin et al. 2013) For example, MyFitnessPal launched in 2005, the Fitbit was introduced in 2009, and the Apple Watch in 2015.
4. Were quantitative research. Grey literature such as theses, conference posters, and unpublished papers were included.
5. Written in English.

Papers were excluded from the review if any of the following applied:

1. Review articles or case studies.
2. Fitness/diet tracking technology was not used in the context of self‐monitoring (i.e., technology was used solely as a measurement approach within a scientific protocol, e.g., Grosser et al. (2020))
3. Articles purely focused on describing the technical design of the FDT.

Given the nascent literature in this area, we included both observational and experimental studies. We use the terminology outlined by Kraemer et al. in the review (1997); observational studies allow us to determine whether FDT use is a correlate of disordered eating, and experimental studies allow us to determine whether FDT use is a causal risk factor for disordered eating and/or ED onset.

### 2.2 Information Sources

The following databases were searched from 1st Jan 2005 to September 5th, 2024: EMBASE, Medline/PubMed, PsychInfo, CINAHL Plus, ERIC, SportDiscus, ASSIA, Social Science Premium, Sociological Abstracts, Sports Medicine and Educational Health, SCOPUS, Cochrane Library, and ProQuest Dissertation and Theses Global. Forward and backward citation chain checking was employed using Google Scholar on the articles included in the final review on the 29th November 2024.

### 2.3 Search Strategy

The following search terms were used:

1. Eating disorder* OR disordered eating OR body image OR body dissatisfaction OR body esteem OR weight concern* OR shape concern OR excessive exercise* OR bulimi* OR anorexi* OR bing* OR orthorexi* OR purg* OR food restrict*
2. Electronic track* OR electronic activ* OR electronic monitor* OR fitness track* OR activity track* OR wearable device* OR wearable act* OR wearable track* OR consumer wearable* OR Fitbit OR SenseWear OR Jawbone OR Nike Fuelband OR Apple Watch OR MyFitnessPal OR Strava OR fitness app* OR calorie track* OR nutrient* track* OR diet track* OR MHealth App*
3. 1 AND 2

### 2.4 Selection Process

Titles and abstracts were screened, followed by a full text review using Covidence (Veritas Health Innovation MA) At least 20% papers were independently screened by a second reviewer. There were 12 disagreements (out of 62 ratings) during title/abstract screening which were resolved through discussion. There were no disagreements during the full‐text review stage. In the case of identifying published and unpublished papers, only published studies were included in the final review.

### 2.5 Data Collection Process

A data extraction form was created and piloted on three papers. Three reviewers independently extracted data for 100% of studies (one extracted data from all studies, while reviewers two and three each extracted 50%); inter‐rater reliability was 94%. Only minor discrepancies emerged which were discussed and resolved.

### 2.6 Data Items

The following information was extracted: title, year of publication, authors, study location, study design, sample size, sample age, sample gender, ethnicity/race, sample body mass index (BMI), clinical or non‐clinical sample, FDT measure(s), disordered eating measure(s), results of relevant tests.

### 2.7 Quality Appraisal of Individual Studies

Three quality assessments from the National Heart, Lung, and Blood Institute (NHLBI) National Heart were used including the Quality Assessment for Observational Cohort and Cross‐Sectional Studies, the Quality Assessment Tool for Before‐After (Pre‐Post) Studies with No Control Group, and the Quality Assessment for Controlled Intervention Studies. Any discrepancies were discussed and resolved.

### 2.8 Synthesis Method

The variance in study design, outcome measures, and statistical analyses rendered a meta‐analysis impossible, and findings were summarized using a narrative synthesis (Popay et al. 2006).

## 3 Results

**FIGURE 1:**
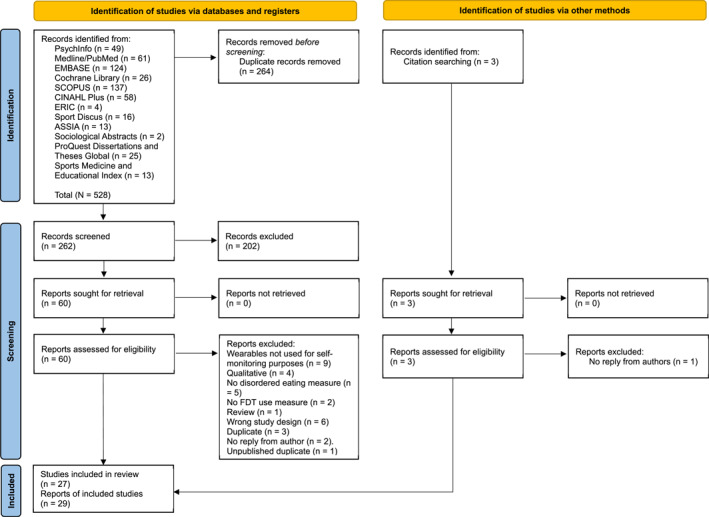
PRISMA flowchart detailing study selection process.

The search returned 262 articles after de‐duplication (Figure 1). Based on title and abstract screening, 202 articles were excluded. The remaining 60 full‐text articles were assessed for eligibility and a further 33 were excluded. Three studies were identified through citation chain searching, one was excluded, resulting in a total of 29 reports from 27 studies (Ajlouni et al. 2023; Asselstine et al. 2023; Berry et al. 2024; Blackstone and Herrmann 2020; Boldi et al. 2024; Elavsky et al. 2017; Smahel et al. 2019; Embacher Martin et al. 2018; Gittus et al. 2020; Guo et al. 2022; Hahn, Kaciroti, et al. 2021; Hahn, Sonneville, et al. 2021; Hahn et al. 2022, 2024; Hefner et al. 2016; Jospe et al. 2018; Kerner et al. 2019; Levinson et al. 2017; Linardon and Messer 2019; Martinelli et al. 2020; Messer et al. 2021; Micanti et al. 2023; O’Loughlin et al. 2023; Plateau et al. 2018; Reynolds et al. 2024; Simpson and Mazzeo 2017; Sun et al. 2023; Tuning 2016; Wallace 2016). Reasons for exclusion can be viewed in Figure 1, with additional information included in the supplementary materials.

### 3.1 Study Characteristics

Publication dates ranged from 2016 to 2024, and the majority of studies were conducted in the USA (*k* = 12) (Table 1). Most studies employed a cross‐sectional survey design (*k* = 17). There were three longitudinal observational studies one collected data from the same participants between 2017 and 2022 (O’Loughlin et al. 2023) and the other two studies utilized the same longitudinal dataset collecting data in 2010 and 2018 (Hahn et al. 2022) with participants invited to participate again in 2022 (Hahn et al. 2024). Five used quasi‐experimental designs: Berry (2024) tested MyFitnessPal over a period of 8 weeks in a group of undergraduate students with no controls; Boldi et al. (2024) tested first‐time Fitbit users over a 4‐month period with a control group; similarly Kerner et al. (2019) tested adolescents after using a Fitbit for 5 weeks; Tuning (2016) compared students who used fitness trackers to a non‐random control group; Martinelli et al. (2020) analysed data from a 12‐week weight loss trial in which FDTs were used. Finally, three studies conducted a randomised controlled trial of FDT use compared to no use, two used a sample of undergraduate students (Gittus et al. 2020; Hahn, Kaciroti, et al. 2021) and one study was conducted in those seeking weight loss treatment (Jospe et al. 2018).

**TABLE 1:** Study characteristics.

Authors	Year	Country	Study design	Type of FDT use	Recruitment setting	Sample size	Mean age in years (SD)	Gender (% female)	Race/ethnicity	Mean BMI (kg/m^2^)
Ajlouni et al.	2023	Jordan	Cross‐sectional	Nutrition and diet apps (type not specified)	Undergraduate students	414	NR	89.9%	NR	NR
Asselstine et al.	2023	USA	Cross‐sectional	Diet apps (type not specified)	Undergraduate student athletes	72	18–23	76.4%	NR	NR
Berry et al.	2024	USA	Quasi‐experimental	MyFitnessPal for 8 weeks	Undergraduate students	68	19.78 (1.48)	100%	56% White 47% Asian	21.9
Blackstone and Herrmann	2020	USA	Cross‐sectional	Fitness trackers (type not specified)	Undergraduate psychology students	337	19.31 (1.56)	100%	82% Caucasian	NR
Boldi et al.	2024	Switzerland	Quasi‐experimental	FitBit use for 4 months	Undergraduate students	321	21.88 (2.81)	61.7%	NR	NR
Elavsky et al. (and Smahel et al.)^a^	2017	Czech Republic	Cross‐sectional	Fitness/diet apps (type not specified)	Websites orientated for eating, exercising and dieting	669 (406)	24.06 (5.23)	84%	92% Czech	22.78
Embacher Martin et al.	2018	United Kingdom	Cross‐sectional	Diet app (type not specified)	Undergraduate students	491	19 (1.26)	52%	64% White	NR
Gittus et al.	2020	Australia	RCT	Fitbit for 10 days	Undergraduate students	262	22.86 (8.47)	100%	NR	22.59
Guo et al.	2022	Mainland China	Cross‐sectional	Fitness/diet tracking app (type not specified)	Undergraduate students	495	19.19 (0.97)	71.3%	96% Han Chinese	20.63
Hahn et al.	2021	USA	RCT	MyFitnessPal for 1 month	Undergraduate students	192	20.2 (2.4)	100%	51% white 29% Latina/Hispanic	21.10
Hahn, Sonneville et al.	2021	USA	Cross‐sectional	Fitness/diet apps or devices (type not specified)^b^	Undergraduate students	647	NR^c^	68.9%	66% Non‐hispanic white 12% Non‐hispanic Asian	23.30
Hahn et al.	2022	USA	Longitudinal	Fitness/diet apps or devices (type not specified)	General public collected for larger study (Project EAT)	1446	*2010*:14.4 (NR) *2018*:22.0 (NR)	59%	28% African American or Black 20% White 20% Asian American	27.20
Hahn, Bornstein et al.^d^	2024	USA	Longitudinal	Fitness/diet apps or devices (type not specified)	General public collected for larger study (Project EAT)	138	26.5 (1.9)	53.3%	31% White 20% Hispanic/Latinx	28.7 (7.7)
Hefner et al.	2016	USA	Cross‐sectional	Fitness/diet apps (type not specified)	Undergraduate students	262	20.48 (1.75)	76%	68% Caucasian 11% Asian	22.63
Jospe et al.	2018	New Zealand	RCT	MyFitnessPal for 15 weeks over 12 months (56 weeks)	Weight loss programme	250	43.67 (10.73)	62%	88% NZEO	33.01
Kerner et al.	2019	United Kingdom	Quasi‐experimental	Fitbit and corresponding app (5 weeks)	School pupils	62	14–15	38.7%	NR	NR
Levinson et al.	2017	USA	Cross‐sectional	MyFitnessPal	Eating disorder clinic	105	25.58 (7.59)	96%	92% European American	NR
Linardon and Messer	2019	Australia	Cross‐sectional	MyFitnessPal	Health and fitness websites	122	28.4 (8.93)	0%	78% Caucasian 11% Asian	26.41
Martinelli et al.	2020	USA	Quasi‐experimental	Fitbit flex and corresponding app	Weight loss programme	77	50.77 (13.39)	80%	53% White/Caucasian 36% Black/African American	34.86
Messer et al.	2021	Australia	Cross‐sectional	Calorie tracker app (type not specified)	General public, wellness social media and forums	1357	30.28 (6.21)	86%	88% White	26.08
Micanti et al.	2023	Italy	Cross‐sectional	Weight‐control app use (type not specified)	Eating disorder clinic	30	20.63 (4.71)	93.3%	NR	20.24
O'Loughlin et al.	2023	Canada	Longitudinal	Food and activity trackers (type not specified)	General public	676	30.5 (1.0)	58.9%	NR	*n* = 12 < 18.5 *n* = 286 18.5 < 24.9 *n* = 281 > 25
Plateau et al.	2018	United Kingdom	Cross‐sectional	Food and activity trackers (type not specified)	Undergraduate students and social media	352	21.90 (3.24)	65%	82% White British	22.87
Reynolds et al.	2024	United Kingdom	Cross‐sectional	Activity tracker/App (type not specified)	School pupils	321	13.55 (0.96)	51.4%	56.6% White British	NR
Simpson and Mazzeo	2017	USA	Cross‐sectional	Fitness and diet app and device (type not specified)	Undergraduate students	493	20.52 (3.64)	69%	50% White 19% Latino/a/Hispanic	24.41
Sun et al.	2023	China	Cross‐sectional	Fitness tracker (WeRun)	General public (WeRun users)	643	31.22 (9.03)	51.6%	NR	NR
Tuning	2016	USA	Quasi‐experimental	Fitness tracker (Nike jawbone)	Undergraduate students	245	17 to 25 (NR)	100%	NR	NR
Wallace	2016	USA	Cross‐sectional	Calorie tracking app (type not specified)	Undergraduate students	175	21.43 (NR)	88%	91% White	NR

Abbreviations: NR = Not Reported, NZEO = New Zealand European and Others, RCT = Randomised Controlled Trial.

^a^ Elavsky et al. (2017) and Smahel et al. (2019) report results from the same sample.

^b^ This included self‐weighing alongside using apps, website, and fitness trackers.

^c^ Range of participants = 18–22 years old.

^d^ Hahn et al. (2024) reports results from a subsample of Hahn et al. (2022) sample.

Sample sizes ranged from *n* = 30 to *n* = 1446, with a total sample size of *N* = 10,584. Mean age ranged from 14.4 to 50.8 years. Five studies had an all‐female sample, one study had an all‐male sample, with the remaining having mixed gender samples (range = 52%–96% female). Twenty‐four studies had general population samples, and four had specialist samples (two involving participants in weight loss trials, and two in people with EDs). Mean BMI in the general population samples ranged from 20.6–28.7 and 33.0–34.9 in the weight loss samples. Only one study in those with EDs reported mean BMI as 20.24 (Micanti et al. 2023).

Regarding the type of FDT, five studies investigated the app MyFitnessPal, four studies investigated Fitbit trackers and the corresponding app, one study investigated the app WeRun, and one study investigated Nike Jawbone fitness trackers. The remaining 16 studies gathered data on the usage of FDTs, however, the type or brand used was not specified.

### 3.2 Quality Appraisal of Individual Studies

The results from the quality appraisal can be viewed in Table 2. Regarding the cross‐sectional and observational assessment (*k* = 21), the majority of these studies clearly outlined their objectives and recruited participants from a similar population. Sixteen studies (76%) did not specify exclusion or inclusion criteria, and only one study provided a justification for their sample size. Additionally, due to the cross‐sectional nature of the majority of studies (*k* = 17; 81%), most did not assess exposure to fitness trackers prior to data collection. Further, due to the lack of a standardised measure to assess FDT use, most studies created their own bespoke measure. There was heterogeneity in the measures used to assess the outcomes with more than 10 different questionnaires being used, the most common being the Eating Disorder Examination Questionnaire (EDE‐Q) (*k* = 7). However, most outcomes were assessed using reliable and valid measures assessing one or more ED outcome. Just over half of studies (*k* = 11, 52%) controlled for at least one potential confounding variable, typically age, race/ethnicity, socio‐economic status or BMI.

**TABLE 2:** Quality assessment of individual studies using NHLBI quality assessment tools.

Quality assessment tool for observational and cross‐sectional studies															
Study	Research question clearly stated	Study population specified and defined	Participation of eligible participants 50%	Recruited from same population	Inclusion/exclusion criteria applied	Sample size justified	Exposure of interest measured prior to outcome measured	Time frame sufficient	Examine different exposure levels	Exposure measure reliable, consistent/valid	Exposure assessed more than once	Outcome measure reliable, valid, consistent	Assessor blind	Loss to follow‐up after baseline ≤ 20%	Consider confounding variables
Ajlouni et al. 2023	+	+	−	+	NR	+	−	−	+	+	−	+	N/A	N/A	−
Asselstine et al. 2023	+	+	−	+	NR	NR	−	−	+	CD	−	+	N/A	N/A	CD
Blackstone 2020	+	+	CD	+	NR	−	−	−	−	−	N/A	+	N/A	N/A	+
Elavsky et al. 2017 and Smahel et al. 2019 ^a^	+	+	CD	+	+	−	−	−	−	−	N/A	+	N/A	N/A	+
Embacher Martin et al. 2018	+	+	−	+	NR	−	−	−	−	−	N/A	+	N/A	N/A	−
Guo et al., 2022	+	+	CD	+	+	−	−	−	−	−	N/A	+	N/A	N/A	+
Hahn, Sonneville et al. 2021	+	+	−	+	+	−	−	−	+	−	N/A	+	N/A	N/A	+
Hahn et al. 2022	+	+	+	CD	NR	−	+	+	−	+	+	+	N/A	+	+
Hahn et al. 2024 ^b^	+	+	−	+	+	−	+	+	+	+	−	+	NR	−	−
Hefner et al. 2016	+	+	CD	+	NR	−	−	−	−	−	N/A	+	N/A	N/A	+
Levinson et al. 2017	+	+	CD	+	NR	−	−	−	−	−	N/A	+	N/A	N/A	−
Linardon et al. 2019	+	+	CD	−	NR	−	−	+	−	−	N/A	+	N/A	N/A	+
Messer et al. 2021	+	−	CD	−	NR	−	−	−	−	−	N/A	+	N/A	N/A	−
Micanti et al. 2023	+	+	−	+	NR	NR	−	−	CD	CD	−	CD	CD	N/A	CD
O'Loughlin et al. 2023	+	+	+	+	NR	−	+	+	+	−	+	−	N/A	+	+
Plateau et al. 2018	+	+	CD	CD	NR	−	−	N/A	+	−	N/A	+	N/A	N/A	−
Reynolds et al. 2024	+	+	CD	+	NR	−	−	N/A	+	+	N/A	+	N/A	N/A	−
Simpson 2017	+	+	CD	+	+	−	−	N/A	−	−	N/A	+	N/A	N/A	+
Sun et al. 2023	+	+	−	+	NR	−	−	N/A	−	+	N/A	+	N/A	N/A	NR
Tuning 2016	+	+	CD	+	NR	−	+	+	−	−	−	+	CD	NR	−
Wallace 2017	+	−	−	+	NR	−	−	N/A	+	−	N/A	+	N/A	N/A	+

Quality assessment tool for before‐after (pre‐post) studies with no control group												
Study	Research question clearly stated	Eligibility criteria specified and defined	Participants representative of target population.	All eligible participants enroled	Sample size sufficient	Intervention clearly described and consistently delivered	Outcome measure reliable, valid, consistent	Outcome assessor's blind to exposure	Loss to follow up ≤ 20% accounted for in analysis	Analysis of outcome measure pre/post intervention	Outcome assessed multiple time pre/post	Analysis accounted for group level interventions
Berry et al. 2024	+	+	+	+	−	+	−	−	+	+	−	N/A
Kerner et al. 2019	+	NR	+	NR	NR	+	+	−	+	+	−	N/A
Martinelli et al. 2020	+	+	+	NR	NR	+	CD	−	+s	+	−	N/A

Quality assessment tool for controlled intervention studies														
	Study described as RT/RCT	Adequate randomisation	Treatment allocation concealed	Participants and providers blinded to group assignment	Outcome assessors blinded to participant group	Baseline characteristics similar at baseline	Overall drop‐out rate ≤ 20% at endpoint	Differential drop‐out at endpoint ≤ 15%	High adherence to intervention protocols	Other interventions avoided	Outcomes measures valid, reliable, applied consistently	Sample size sufficient to detect group differences	Prespecified outcomes/subgroup analysis	Randomised participants analysed in original group
Boldi et al. 2024	+	−	−	−	−	−	+	+	NR	+	+	−	+	+
Gittus et al. 2020	+	−	−	−	−	−	+	+	NR	+	+	−	+	+
Hahn et al. 2021	+	+	+	−	−	CD	+	+	+	+	+	+	+	+
Jospe et al. 2018	+	+	+	−	−	CD	−	−	−	−	+	−	+	+

*Note:* CD = Cannot Determine, + = Yes, − = No, N/A = Not Applicable, NR = Not reported.

^a^ Elavsky et al. (2017) and Smahel et al. (2019) report results from the same sample.

^b^ Hahn et al. (2024) reports results from a subsample of Hahn et al. (2022) sample.

Of the three studies assessed using the quality assessment tool for before‐after studies with no control group (Berry et al. 2024; Hahn et al. 2024; Kerner et al. 2019), all studies pre‐specify their eligibility criteria and clearly explained their intervention. Additionally, no study reported a loss to follow‐up greater than 20% and accounted for loss to follow‐up in their analyses. However, it was unclear if any study had a sufficient sample size and no study used a reliable measure to assess outcomes or blinded the assessor to the participants exposure to the outcome. Finally, results of the quality assessment of the controlled intervention studies revealed that two out of four studies had inadequate randomization and treatment allocation was not concealed (Boldi et al. 2024; Gittus et al. 2020). Further, one study conducted by Jospe et al. reported a loss to follow‐up of greater than 20%, as well as a differential drop‐out rate greater than 15%, and a low adherence to intervention protocols (Jospe et al. 2018). Finally, only one study had a sufficient sample size to detect group differences (Hahn, Kaciroti, et al. 2021).

### 3.3 Synthesis of Findings

Study results are given in Table 3 (observational studies) and Table 4 ([quasi‐]experimental studies). Syntheses of findings are presented first for general population samples, separated by disordered eating outcome. As very few studies focused specifically on adolescent populations, we present all age groups together. Where differences emerged by gender, we report these. Next, we present findings for specialist populations (those with EDs and those in weight loss programmes). To highlight the level of evidence available for each outcome, we first present observational studies followed by experimental studies.

**TABLE 3:** Individual study results of observational studies by disordered eating outcome.

Author/year	Global disordered eating	Dietary restraint	Shape and weight concerns	Binge eating	Compensatory behaviours	Excessive exercise	Disordered muscle‐orientated behaviour
Ajlouni et al. 2023	—	—	mHealth app use correlated with positive body image (*r* = 0.289)	—	—	—	—
Asselstine et al. 2023	There was no significant association between the number of athletes who screened positive for disordered eating and used a diet tracking app. Frequency of diet app use did not correlate with disordered eating.	—	—	—	—	—	—
Blackstone 2020	—	—	—	—	—	Wearable users reported higher exercise dependence compared with non‐users (*β* = 0.116).	—
Elavsky et al. 2017 and Smahel et al. 2019	—	—	App users had higher drive for thinness than non‐app users, though this was non‐significant when controlling for potential confounding variables (e.g., gender, age). Higher drive for thinness was associated with using apps for weight monitoring (*β* = 0.26), planning and determining state of health (*β* = 0.13). But not for socializing functions (*β* = 0.02), counting steps, monitoring sleep or heart rate.	—	—	App users had higher excessive exercise scores than non‐app users, even when adjusting for potential confounding variables (e.g., gender, age). Excessive exercise was a predictor of using apps for weight and monitoring (*β* = 0.12), socializing (*β* = 0.16), and planning and monitoring goals (*β* = 0.25), but not with counting steps, monitoring sleep and health data.	—
Embacher Martin et al. 2018	—	—	Users of calorie tracking apps had higher body dissatisfaction than non‐users. Body dissatisfaction had a direct effect on calorie app usage (*β* = 0.28) and mediated the relationship between gender and calorie tracking (*β* = 0.08)	—	—	—	—
Guo et al., 2022	Weight and fitness app use correlated with disordered eating for females (*r* = 0.49) but not for males (*r* = 0.12). Disordered eating cognition mediated the relationship between weight and fitness app use and disordered eating behaviours.	Weight and fitness app use correlated with dietary restraint for females (*r* = 0.48) but not for males (*r* = 0.18).	Weight and fitness app use correlated with shape concern for females (*r* ≤ 0.46). But not for males (*r* ≤ 0.15.	Weight and fitness app use correlated with binge eating for females (*r* = 0.23) but not males (*r* = 0.18).	Weight and fitness app use did not correlate with self‐induced vomiting or laxative misuse for females (*r* ≤ 0.13) or males (*r* ≤ 0.16).	Weight and fitness app use correlated with excessive exercise for females (*r* = 0.35) but not for males (*r* = 0.22).	—
Hahn, Sonneville et al. 2021	—	Participants who used weight and exercise related self‐monitoring tools were more likely to fast and skip meals compared to those who used minimal self‐monitoring. Males who used exercise related self‐monitoring only were less likely to skip meals compared to those who used both fitness and weight related self‐monitoring tools.	—	—	Females who used weight and exercise related self‐monitoring tools were not more likely to engage in purging and appetite suppressing compared to those who used minimal self‐monitoring. Males who used weight and exercise related self‐monitoring only, were more likely to engage in purging and appetite suppressing compared to those who used minimal self‐monitoring.	Females (but not males) who used weight and exercise related self‐monitoring tools were more likely to engage in excessive exercise compared to those who used minimal self‐monitoring.	—
Hahn et al. 2022	Using diet‐ or activity‐focused apps in adolescence was longitudinally associated with greater disordered eating behaviours in early adulthood	—	—	—	—	—	Using diet‐ or activity‐focused apps in adolescence was longitudinally associated with greater disordered muscle building behaviours in early adulthood.
Hahn et al. 2024	The type of tracker used (diet, fitness, or any app) wasn't longitudinally associated with global disordered eating. Using apps for weight management motivations but not but not healthy eating or physical activity was associated with an increase in global disordered eating over time.	—	—	The type of tracker used (diet, fitness, or any app) wasn't longitudinally associated with binge eating. Using apps for weight management, health eating or physical activity motivations had no longitudinal association with binge eating.	—	The type of tracker used (diet, fitness, or any app) wasn't longitudinally associated with compulsive exercise. Using apps for weight management, health eating or physical activity motivations had no longitudinal association with compulsive exercise.	The type of tracker used (diet, fitness, or any app) wasn't longitudinally associated with disordered muscle building behaviour. Using apps for weight management, health eating or physical activity motivations had no longitudinal association with disordered muscle building behaviour.
Hefner et al. 2016	Using fitness and diet apps was associated with higher disordered eating (*β* = 0.34).	—	—	—	—	Using fitness and diet apps was associated with higher compulsive exercise (*β* = 0.31).	—
Levinson et al. 2017	—	People with EDs who reported calorie tracking contributed to their ED symptoms scored higher on dietary restraint (*r* = 0.25).	People with EDs who reported calorie tracking contributed to their ED symptoms scored higher on shape and weight concern (*r* ≤ 0.36).	—	—	—	—
Linardon et al. 2019	MyFitnessPal users reported significantly higher disordered eating scores compared to non‐users (*d* = 0.91). After controlling for potential confounding variables (e.g., ED behaviours, demographics), MyFitnessPal was a significant predictor of ED symptoms (*β* = −0.15).	MyFitnessPal users reported significantly higher dietary restraint compared to non‐users (*d* = 1.06).	MyFitnessPal users reported significantly higher shape and weight concerns compared to non‐users (*d* ≤ 0.79).	MyFitnessPal users reported significantly more binge eating episodes compared to non‐users (*r* = − 0.35).	There were no differences between MyFitnessPal users and non‐users in rates of compensatory behaviours (*r* = − 0.09).	—	—
Messer et al. 2021	Those who reported ever using a calorie‐tracking app had significantly higher disordered eating scores compared to non‐users (*d* = 0.94). Longer duration of app use was correlated with higher disordered eating scores (*r* = 0.18). Those who used apps for weight and shape reasons had higher disordered eating scores compared to those who used apps for health reasons (*d* = 0.74).	—	—	There were no differences in binge eating frequency between users and non‐users of calorie‐tracking apps (*d* = 0.04). Duration of app use was unrelated to binge eating frequency (*r* = 03). There were no differences in binge eating frequency between those using tracking apps for weight and shape reasons compared to health reasons (*d* = 0.02).	Those who reported ever using a calorie‐tracking app reported more frequent compensatory behaviours compared to non‐users (*d* = 0.26). Longer duration of app use was correlated with compensatory behaviour frequency (*r* = 0.15). Those who used apps for weight and shape reasons reported more compensatory behaviours compared to those who used the app for health reasons (*d* = 0.42).	—	Those who reported ever using a calorie‐tracking app reported higher muscularity‐oriented disordered eating compared to non‐users (*d* = 0.1.17). Longer duration of app use was correlated with greater muscularity‐oriented disordered eating (*r* = 0.23). Those who used apps for weight and shape reasons reported higher muscularity‐oriented disordered eating compared to those who used apps for health reasons (*d* = 0.62).
Micanti et al. 2023	66% (*n* = 20) of participants with an ED diagnosis reported an increase in use of fitness (and social media) apps.	—	—	—	—	—	—
O'Loughlin et al. 2023	Having a diagnosis of an ED predicted past year activity (OR = 5.3) and food tracking (OR = 3.3), however this relationship was not significant for food tracking after adjusting for confounding variables.	—	—	—	—	—	—
Plateau et al. 2018	—	FDT users reported higher dietary restraint than non‐users (*r* = 0.26). Higher frequency of use of activity monitoring (*r*_*s*_ = ≥ 0.17) (but not food monitoring, *r*_*s*_ ≤ 0.13) was associated with greater dietary restraint. Using activity monitoring (but not food monitoring) for weight and shape related reasons was associated with greater dietary restraint than using FDTs for wellbeing or fitness reasons (*η*_*p*_^2^ = 0.05).	FDT users reported greater weight concern (*r* = 0.16), but not greater shape concern (*r* = 0.10) compared with non‐users. Higher frequency of use of activity monitoring (*r*_*s*_ = ≥ 0.17) (but not food monitoring, *r*_*s*_ ≤ 0.13) was associated with greater weight concern (but not shape concern). Using FDTs for weight and shape related reasons was associated with greater weight and shape concerns than using FDT for wellbeing or fitness reasons for both activity monitoring (*η*_*p*_^2^ = 0.09) and food intake monitoring (*r* ≤ 0.33)	There were no significant differences between FDT users and non‐users in rate of binge eating (V = 0.07). Frequency of FDT use was unrelated to rate of binge eating. Motivations for FDT use were unrelated to rate of binge eating.	FDT users reported more purging than non‐users (V = 0.19). Frequency of FDT use was unrelated to rate of purging. Using FDTs for weight and shape related reasons was associated with a higher rate of purging behaviours than using FDTs for wellbeing or fitness reasons (V = 0.32).	FDT users reported higher compulsive exercise in some aspects (weight control (*r* = 0.48), mood improvements (*r* = 0.27) but not others (avoidance (*r* = 0.17), lack of enjoyment (*r* = 0.05) rigidity (*r* = 0.18)) compared to non‐users. Higher frequency of use of activity monitoring (but not food monitoring, *r*_*s*_ ≤ 0.13) was associated with greater exercise for weight control reasons (*r*_*s*_ = ≤ 0.19) but not other aspects of compulsive exercise. Using FDTs for weight and shape related reasons was associated with greater exercise for weight control reasons (activity monitoring: *η*_*p*_^2^ = 0.12, food intake monitoring: *r* = 0.40) but not other aspects of compulsive exercise (activity monitoring: *η*_*p*_^2^ ≤ 0.04 food intake monitoring: *r* ≤ 0.11).	—
Reynolds et al. 2024	—	—	—	—	—	Exercise monitoring was unrelated to any aspect of compulsive exercise. Other aspects of app/device use including feelings of obligations for tracking predicted facets of compulsive exercise including avoidance (*β* = 0.42), lack of enjoyment (*β* = 0.22), and exercise rigidity (*β* = 0.26) but not weight control or mood improvement (*p* = n.s.) Using an exercise app/device for fitness guidance was associated with the following facets of compulsive exercise, avoidance (*β* = 0.17), compulsive mood improvement (*β* = 0.32), and exercise rigidity (*β* = 0.25) but not weight control or lack of enjoyment. Comparing exercise tracking with others was associated with compulsive exercise weight control only (*β* = 0.18)	—
Simpson 2017	Use of fitness tracking apps (but not calorie tracking apps, *β* = 0.04) was associated with greater disordered eating (*β* = 0.12).	Use of calorie tracking apps (*η* ^2^ = 0.03) (but not fitness tracking apps, *η* ^2^ ≤ 0. 001) was associated with higher dietary restraint.	Neither use of calorie nor fitness tracking apps (*η* ^2^ ≤ 0. 001) was related to weight and shape concerns.	—	—	—	—
Sun et al. 2023	Using a fitness app was positively associated with body dissatisfaction (*β* = 0.25)	—	—	—	—	—	—
Wallace (2016)	Use of calorie tracking apps was associated with greater disordered eating (*β* = 0.43).	—	—	—	—	—	—

*Note:* ‘—’ indicates a specific outcome was not measured by the study. Effect sizes have been reported where possible.

Abbreviations: ED = Eating disorder, FDT = Fitness and Diet Tracker.

**TABLE 4:** Individual study results of experimental and quasi‐experimental studies by disordered eating outcome.

Author/Year	Global disordered eating	Dietary restraint	Shape and weight concerns	Binge eating	Purging	Excessive exercise
Berry et al. 2024	—	Average levels of dietary restraint over the study period were unrelated to frequency of fitness tracking. Higher frequency of tracking at one time point predicted increased dietary restraint at the following time point within a day (but not across days). Baseline levels of ED symptoms, perfectionism and rumination about food did not moderate the association between tracking and dietary restraint.	Average levels of weight and shape concerns (but not body dissatisfaction) were associated with higher frequency of fitness tracking over the study period. Higher frequency of tracking at one timepoint/day was unrelated to changes in weight and shape concerns or body dissatisfaction at the next time point/day Baseline levels of ED symptoms, perfectionism and rumination about food did not moderate the association between tracking and weight and shape concerns/body dissatisfaction.	—	—	Frequency of tracking was unrelated to obligatory exercise (overall, within and between days). Baseline levels of ED symptoms, perfectionism and rumination about food did not moderate the association between tracking and obligatory exercise.
Boldi et al. 2024	—	—	There were no differences in body image in men or women compared to non‐users after 4 months of use.	—	—	—
Gittus et al. 2020	—	Fitbit users were less likely to report dietary restraint compared to controls	There were no differences in levels of body satisfaction between Fitbit users and controls	Fitbit users were less likely to report binge eating compared to controls	—	There were no differences in levels of exercise dependence between Fitbit users and controls
Hahn et al. 2021	There was no difference between MyFitnessPal users and non‐users in disordered eating scores after 1 month of use (*β* = − 0.04).	There was no difference between MyFitnessPal users and non‐users in dietary restraint after 1 month of use (fasting OR = 0.47; limiting intake OR = 0.55).	—	There was no difference between MyFitnessPal users and non‐users in binge eating behaviours after 1 month of use (OR = 1.51).	No participants reported purging.	There was no difference between MyFitnessPal users and non‐users in compulsive exercise after 1 month of use (OR = 0.61).
Kerner et al. 2019	—	—	After using a Fitbit for 5 weeks participants had significantly lower reports of body dissatisfaction (*η* ^2^ = 0.08)	—	—	—
Tuning 2016	—	—	—	—	—	There were no differences in levels of compulsive exercise between those who used the jawbone for a semester when compared with controls who did not (*β* = −0.14).
Jospe et al. 2018	There were no differences in disordered eating scores at 12 months follow up between those allocated to use MyFitnessPal for 15 weeks compared to no self‐monitoring.	There were no differences in dietary restraint at 12 months follow up between those allocated to use MyFitnessPal for 15 weeks compared to no self‐monitoring.	There were no differences in shape and weight concerns at 12 months follow up between those allocated to use MyFitnessPal for 15 weeks compared to no self‐monitoring.	There were no differences in rates of binge eating at 12 months follow up between those allocated to use MyFitnessPal for 15 weeks compared to no self‐monitoring.	Rates of purging were too low to test group differences.	There were no differences in excessive exercise at 12 months follow up between those allocated to use MyFitnessPal for 15 weeks compared to no self‐monitoring.
Martinelli et al. 2020	—	—	—	Binge eating severity prior to treatment predicted greater self‐monitoring of weight and eating (*r*_*s*_ = 0.25), but not activity (*r*_*s*_ = 0.08) during the trial period.	—	—

*Note:* No studies assessed disordered muscle orientated behaviour. ‘—’ indicates a specific outcome was not measured by the study. Effect sizes have been reported where possible.

Abbreviations: ED = Eating disorder, FDT = Fitness and Diet Tracker.

#### 3.3.1 Global Disordered Eating

Overall, 10 observational studies reported on a global measure of disordered eating (e.g., EDE‐Q, EAT‐26), with relatively consistent findings of FDT use being positively correlated with disordered eating (Asselstine et al. 2023; Guo et al. 2022; Hahn et al. 2022, 2024; Hefner et al. 2016; Linardon and Messer 2019; Messer et al. 2021; Simpson and Mazzeo 2017; Sun et al. 2023; Wallace 2016). Seven of eight cross sectional studies reported greater disordered eating in FDT users compared with non‐users with small to large effect sizes (Guo et al. 2022; Hefner et al. 2016; Linardon and Messer 2019; Messer et al. 2021; Simpson and Mazzeo 2017; Sun et al. 2023; Wallace 2016). The one cross sectional study that found no association between FDT use and global disordered eating specifically recruited athletes (Sun et al. 2023). Building on the relatively consistent cross‐sectional findings, one longitudinal study showed that FDT use in adolescence was prospectively associated with increases in disordered eating in early adulthood (Hahn et al. 2022).

Regarding potential mechanisms explaining the association, there is some evidence that the association between FDT use and disordered eating was mediated by disordered eating cognitions (Guo et al. 2022). Other studies have explored whether specific types of FDT (e.g., diet vs. fitness) were differentially associated with disordered eating (Hahn et al. 2024; Simpson and Mazzeo 2017). However, no clear picture emerges. For example, Simpson and Mazzeo (2017) found that fitness tracker but not calorie tracker usage was associated with disordered eating behaviours, but this result has not been replicated elsewhere (Hahn et al. 2024). There are also mixed findings regarding gender; Guo et al. (2022) reported significant associations in women only, while other studies found no difference between genders (Hahn et al. 2024) or significant associations in male‐only samples (Linardon and Messer 2019).

Only one experimental study explored the impact of assigned FDT use on global disordered eating in the general population (Hahn, Kaciroti, et al. 2021) and reported no difference in disordered eating rates between those assigned to FDT use versus controls.

#### 3.3.2 Dietary Restraint

Five cross‐sectional studies found evidence of a concurrent association between FDT use and dietary restraint in the general population (Guo et al. 2022; Hahn, Sonneville, et al. 2021; Linardon and Messer 2019; Plateau et al. 2018; Simpson and Mazzeo 2017) with small to large effect sizes. Collectively, these studies suggested that FDT users tended to concurrently engage in greater dietary restraint than non‐users.

Some evidence suggested that the type of FDT used and motivations for FDT use may moderate these associations, although the picture is complex: Simpson and Mazzeo (2017) reported that dietary restriction was associated with calorie tracker, but not fitness tracker use. Plateau et al. (2018) found that using activity‐monitoring tools (but not food‐monitoring tools) for weight and shape concern reasons specifically was associated with higher dietary restraint.

Findings from three experimental/quasi‐experimental studies (Berry et al. 2024; Gittus et al. 2020; Hahn, Kaciroti, et al. 2021) were less consistent and were contradictory to the cross‐sectional studies. Berry (2024) reported that, whilst average levels of dietary restraint over the study period were unrelated to fitness tracking, higher levels of fitness tracking at a particular time‐point were associated with higher dietary restraint intentions at the following time‐point in that day. In contrast, Hahn, Kaciroti, et al. (2021) found no differences in the levels of fasting in those who used MyFitnessPal for a month versus those who did not, whereas Gittus et al. (2020) found that those allocated to use a Fitbit for 10 days endorsed significantly *lower* levels of dietary restraint than non‐users.

#### 3.3.3 Weight and Shape Concerns

Seven observational studies examined the association between FDT use and weight and shape concerns, with inconsistent findings (Ajlouni et al. 2023; Elavsky et al. 2017; Embacher Martin et al. 2018; Guo et al. 2022; Linardon and Messer 2019; Plateau et al. 2018; Simpson and Mazzeo 2017). For example, Simpson and Mazzeo (2017) reported no association between FDT use and weight and shape concerns. Ajlouni et al. (2023) conversely reported that greater mHealth app use was correlated with *more positive* body image. Other studies reported the converse, that is, significant relationships between FDT use and higher levels of weight and shape concerns or drive for thinness with small to medium effect sizes (Embacher Martin et al. 2018; Guo et al. 2022; Linardon and Messer 2019; Plateau et al. 2018). Mixed findings here might be explained by differing frequency and motivations for use; Plateau et al. (2018) found that weight and shape concern were significantly higher for those who used apps for weight and shape reasons, rather than for health and well‐being or fitness goal reasons. Similarly, more frequent use of activity monitoring (but not food monitoring) was associated with greater weight concerns.

Four experimental/quasi‐experimental studies explored the impact of assigned FDT use on weight and shape concerns, again with very mixed findings (Berry et al. 2024; Boldi et al. 2024; Gittus et al. 2020; Kerner et al. 2019). Boldi et al. (2024) and Gittus et al. (2020) reported no impact of FDT use on weight and shape concerns whilst Kerner et al. (2019) found an increase in body satisfaction after FDT use. In contrast, Berry (2024) reported that average levels of weight and shape concerns over the study period were higher in participants who engaged in more frequent fitness tracking. But higher levels of fitness tracking at a particular timepoint, or on particular day, were unrelated to changes weight and shape concerns (at the next time point/following day). In the same sample, body dissatisfaction was unrelated to fitness tracking in all analyses.

#### 3.3.4 Binge Eating

Five observational studies investigated binge eating and FDT use with mixed findings (Guo et al. 2022; Hahn et al. 2024; Linardon and Messer 2019; Messer et al. 2021; Plateau et al. 2018). One cross‐sectional study reported no differences in binge eating between app‐users and non‐users (Plateau et al. 2018), whereas others reported more binge eating in app users, although there are inconsistencies in whether this was in men (Linardon and Messer 2019) or women (Guo et al. 2022). One longitudinal study found that neither app type (i.e., dietary‐focused or activity‐focus vs. no app use) or motivation for use predicted future binge eating behaviour (Hahn et al. 2024).

Two experimental studies explored how FDT use related to binge eating, again with conflicting results. Gittus et al. (2020) reported that participants allocated to use Fitbits for 10 days were *less* likely to engage in binging than non‐users. In comparison, Hahn, Kaciroti, et al. (2021) reported no differences in binge eating rates for those assigned to use MyFitnessPal for 1 month compared with non‐users.

#### 3.3.5 Compensatory Behaviours

Five observational studies investigated the associations between FDT use and compensatory behaviours (Guo et al. 2022; Hahn, Sonneville, et al. 2021; Linardon and Messer 2019; Messer et al. 2021; Plateau et al. 2018), again with mixed findings. Whilst two of these studies reported no association (Guo et al. 2022; Linardon and Messer 2019), three found that FDT users engaged in significantly more compensatory behaviours compared with non‐users (Hahn, Sonneville, et al. 2021; Messer et al. 2021; Plateau et al. 2018). Messer et al. (2021) and Plateau et al. (2018) both reported higher rates of purging in participants using FDTs for weight and shape reasons compared to health and fitness reasons. All effect sizes were small. One experimental study looked at the effect of FDT use on purging behaviours (Hahn, Kaciroti, et al. 2021), but the frequency of purging behaviours was too low for the impact to be determined.

#### 3.3.6 Excessive Exercise

Seven observational studies (Blackstone and Herrmann 2020; Elavsky et al. 2017; Guo et al. 2022; Hahn, Sonneville, et al. 2021; Hefner et al. 2016; Plateau et al. 2018; Reynolds et al. 2024) investigated the association between FDTs use and excessive exercise or related constructs. All reported at least one significant positive association between FDT use and higher levels of excessive exercise, compulsive exercise or exercise dependence compared with non‐use with small to medium effect sizes. Plateau et al. (2018) additionally reported that greater frequency of activity monitoring, and using FDTs for weight and shape concern reasons, were associated with higher rates of exercise for weight‐control reasons. In terms of higher‐risk groups, two studies found an association between FDT use and excessive exercise in females but not males (Guo et al. 2022; Hahn, Sonneville, et al. 2021). In contrast, all four experimental/quasi‐experimental studies looking at the impact of FDT use on excessive exercise found no change after onset of FDTs use/associations with tracking frequency (Berry et al. 2024; Gittus et al. 2020; Hahn, Kaciroti, et al. 2021; Tuning 2016).

#### 3.3.7 Disordered Muscle‐Orientated Behaviour

Finally, only two studies investigated the association between FDTs use and disordered muscle‐orientated behaviour (e.g., androgenic anabolic steroid use) (Hahn et al. 2022; Messer et al. 2021). Both found evidence supporting an association between FDT use and higher disordered muscle‐oriented behaviours. Messer et al. (2021) reported that calorie‐tracking app users endorsed significantly higher scores on the muscle‐orientated eating test, with those using apps for weight and shape reasons exhibiting significantly higher scores compared to those using the app for health reasons (with large and medium effect sizes respectively). Hahn et al. (2022) found a longitudinal association between diet‐ or fitness‐focused app use in adolescence and increased disordered muscle‐orientated behaviour in young adulthood. However, in contrast to Messer's findings, neither app type (e.g., fitness or diet vs. no app use) nor motivations for use predicted disordered muscle‐orientated behaviour (Hahn et al. 2024). No experimental studies explored the impact of FDTs on disordered muscle‐orientated behaviours.

#### 3.3.8 Specialist Populations

Five studies investigated specialist populations; three included clinical samples of participants who had been diagnosed with an ED (Levinson et al. 2017; Micanti et al. 2023; O’Loughlin et al. 2023) and two included weight‐loss intervention samples (Jospe et al. 2018; Martinelli et al. 2020).

O'Loughlin (2023) reported that those who had an ED diagnosis were five times more likely use activity trackers in the past year compared with those without an ED diagnosis, but no more likely to use diet trackers (once demographic characteristics were adjusted for). Micanti et al. (2023) examined fitness and social media app use in participants with an ED, reporting that 66% (*n* = 20) increased their app use during the COVID‐19 pandemic. Finally, Levinson et al. (2017) asked participants how much they felt MyFitnessPal contributed to their ED. The study then explored the relationship between these ratings and scores on the EDE‐Q. Of 78 users of MyFitnessPal, 83% stated that the app ‘at least somewhat contributed’ to their ED, with 30.3% stating it ‘very much contributed’ to their ED. Further analysis revealed that both dietary restraint and weight and shape concerns were significantly higher in participants who endorsed that using MyFitnessPal had contributed to their ED with small and medium effect sizes respectively.

Regarding weight loss treatment samples, Martinelli et al. (2020) reported that participants with higher binge eating severity prior to weight loss treatment engaged in greater self‐monitoring of eating using FDTs during the weight loss programme. However, this effect was not found for fitness tracking. Jospe et al. (2018) conversely found no significant differences in disordered eating scores between those allocated to use MyFitnessPal intermittently as part of a weight loss programme over 12 months compared to controls.

## 4 Discussion

This systematic review aimed to investigate the association between FDT use and disordered eating behaviours across different populations, including clinical groups. The studies identified showed a nascent but growing area of research on this topic. Overall, there was reasonably consistent evidence supporting a cross‐sectional association between FDT use and some disordered eating behaviours; specifically, FDT users tend to report greater overall disordered eating pathology, dietary restraint, excessive exercise and disordered muscle‐oriented behaviours compared with non‐users (although these differences tend not to be large). Importantly, these associations were not replicated in experimental studies where participants are instructed to use FDTs for a set period. Therefore, based on the available evidence, whilst FDT use seems to be a correlate of some facets of disordered eating, it is not possible to conclude that FDT use causes an increase in disordered eating behaviours.

Given that many people who opt to start using FDTs report doing so to support desired changes in body weight and shape (Hahn et al. 2024; Messer et al. 2021), it is perhaps not surprizing that people who use FDTs are more likely to report reducing food intake and increasing exercise behaviours (i.e., behaviours are commonly believed to underpin body composition change). The dearth of longitudinal evidence available means that it is not yet clear whether naturalistic FDT use is simply a marker of another underlying risk factor for disordered eating behaviours (e.g., increased drive for thinness), or whether the FDT use itself has a mechanistic role to play as a risk factor (e.g., through encouraging dieting). The possibility of a bi‐directional relationship is also worth considering; it is feasible that individuals experiencing weight and shape concerns, or disordered eating are more drawn to FDTs, and that continued FDT use could exacerbate these difficulties over time. Given the limited longitudinal and experimental research, it would be helpful to explore the nature of the temporal association further. This research is important as understanding the extent to which FDT use is a marker versus risk factor for disordered eating has implications for whether intervention in FDT use is likely to improve outcomes.

In contrast to the cross‐sectional studies, the experimental findings in the current review generally show a lack of impact of FDT use on disordered eating outcomes. In this sense, the early experimental literature is promising regarding the safe use of these devices in the general population. However, there were several note‐worthy limitations to the experimental studies, which should caution strong conclusions and instead point to the need for more research in this area, particularly for potentially vulnerable groups. Importantly, the experimental work tends to focus on shorter term (i.e., several weeks) of directed use, and tend to specifically exclude people at higher risk for EDs (Hahn, Kaciroti, et al. 2021). We therefore know very little about the potential impact of longer term, more naturalistic use, on those who may be more vulnerable and opt to spontaneously use FDTs.

It is feasible that there are differences in the associations between FDT use and disordered eating for specific products or features (e.g., types of data recorded and displayed, nature of notifications/prompts, nature of connection with others), which we were unable to explore in this review. Those studies that have begun to explore the facets of FDT use that might be associated with better/worse outcomes give early indication that greater frequency of use and using apps for the purpose of changing weight and shape may be more strongly associated with disordered eating (Messer et al. 2021; Plateau et al. 2018). This aligns with previous research showing that extrinsic motivations for exercise are linked with disordered eating symptoms, such as excessive exercise, drive for thinness, and binge eating (Staples et al. 2022; Verstuyf et al. 2016). Future work exploring specific features and motivations for use in more detail is needed before recommendations about how to maximize any benefit and minimize any harm can be made.

Regarding specialist populations, the review highlights a clear dearth of literature focused on the impact of FDTs in people with clinical EDs (especially binge‐related EDs). The very limited evidence presented here suggests that diet and fitness trackers are commonly used by, and may pose particular challenges for, those with EDs. This aligns with qualitative work where participants with an ED diagnosis reported setting unrealistic targets to reduce their caloric intake and obsessively using the app (Smahelova et al. 2020). As food/calorie and activity tracking can be a core feature of many EDs, whether FDT use exacerbates symptoms, or simply reflects symptom severity, remains unknown. It is worth highlighting that dietary tracking does form parts of some ED therapies (e.g., using food diaries as part of cognitive behavioural therapy for eating disorders and behavioural weight management in binge eating disorder (Masheb et al. 2011; Grilo et al. 2020)). However, FDTs, which provide instant feedback, direct comparisons with other users and gamification of behaviours, seem to offer a very different tracking experience to those used within psychological therapies. Further work is needed to explore this important area and the potential for safe use of these technologies within healthcare.

Finally, as the research identified in this review was limited to predominantly white, female, undergraduate students with a BMI below 30, there are many other potentially vulnerable groups which need to be considered for future research. For example, no study analysed the possible effects of other important demographic features that are known to be linked to EDs, such as being from a racial/ethnic, gender or sexual minority (Parker and Harriger 2020; Raney et al. 2023). Additionally, very little work has specifically explored the impact of FDTs in adolescent populations, which is an important omission given the rising popularity of this technology in younger groups and the high risk for the onset of EDs and other mental health conditions during this developmentally sensitive period (Solmi et al. 2022). Future research here is needed; understanding any mechanisms that may drive particular groups to experience negative outcomes from FDT use will be an important precursor to developing preventative interventions for those at increased risk.

### 4.1 Strengths and Limitations

This review is the first to systematically examine the relationship between FDT use and disordered eating and identifies important trends and gaps in the literature. Whilst we have used rigorous systematic review methodology, the heterogeneous study designs precluded a meta‐analysis. The study samples primarily comprised of women aged 19–25 years with a BMI between 18.5 and 30.0, reducing the generalizability of the review findings and limiting current evidence on FDT use in low weight, specialist or more diverse populations. We also included only English language papers in our review. Whilst no identified papers were excluded on the grounds of being written in a non‐English language, it may be that our search strategy missed relevant research from non‐anglophone research groups.

### 4.2 Recommendations

Given the limitations of the evidence base presented above, firm conclusions about the use of FDTs in the context of disordered eating cannot yet be made. Cross‐sectional evidence does suggest that FDT use may be a marker of an underlying risk for disordered eating in some vulnerable groups, and so some caution may be warranted. Given that FDTs may be used to support evidence‐based behaviour change techniques (Cheng Chia et al. 2019), for example, self‐monitoring in weight‐loss interventions (Carpenter et al. 2022; Carter et al. 2013; Patel et al. 2019), there is the potential that these technologies may facilitate *positive* outcomes in some groups.

In terms of future research, in addition to understanding how mechanisms of FDTs may contribute to or maintain disordered eating, exploration of how, for whom, and under what circumstances FDTs may be used to support positive behaviour change is an important consideration. Detailed research exploring whether there are particular features that are more or less strongly associated with disordered eating would also help to guide salutogenic developments in this field. As FDT technology continues to develop rapidly, any review will also need regular updating to be relevant for contemporary devices.

### 4.3 Conclusion

The current review reveals a cross‐sectional association between the use of FDTs and some aspects of disordered eating. More research is needed to understand the direction and causality of the relationship between FDT use and disordered eating practices, specifically regarding populations that may be at increased risk. Whilst it is likely that many people can use FDTs without experiencing disordered eating difficulties, enquiring about FDT use in clinical contexts (e.g., people with EDs) is important in order to explore how and why FDTs are being used. Ultimately, further research on FDT use and its impact is needed to underpin intervention development to minimize risk and maximize positive outcomes from these technologies.

## Author Contributions

conceptualization (L.R. and H.S.), data curation (S.M. and L.R.), formal analysis (S.M., M.‐C.O., C.B., D.D. and V.L.), writing original draft (S.M., L.R., M.‐C.O. and H.S.), writing review and editing (S.M., M.‐C.O., A.H., B.I., C.B., C.K., D.D., M.P., U.S., and H.S.).

## Ethics Statement

The authors have nothing to report.

## Consent

The authors have nothing to report.

## Conflicts of Interest

The authors declare no conflicts of interest.

## Supporting information

Table S1

## Data Availability

Data sharing is not applicable to this article as no new data were created or analysed in this study.
