# Supplementary material for: Associations Between the Use of Fitness and Diet Tracking Technology and Disordered Eating Behaviour: A Systematic Review
Source: Eur Eat Disord Rev. 2025 Jul 10;33(6):1288–313. doi: 10.1002/erv.70006 (PMC12547374; doi:10.1002/erv.70006)
Supplement: Supplementary file 1 — Table S1 [file ERV-33-1288-s001.docx]

**Supplementary Material**

**S1. Excluded Studies – Further information on reasons for exclusion.**

| **Table S1.** *Excluded Studies and further clarification on reasons for exclusion.* | | |
| --- | --- | --- |
| Reference | Reason for Exclusion | Further Clarification |
| Alexander, R.C., Harper-Cooks, T., Robles, J., Campos, A.P., Matthes, K.E., Palacios, J.E. & Goode, R.W. (2024) Examining the mHealth Needs for Nutrition Monitoring in Black Women Who Binge Eat. *Current Developments in Nutrition, 8(2).* | Wrong study design | Although participants reported experiencing disordered eating behaviours (binge eating) and using a nutrition monitoring app (Nourishly). This app is designed to be used in collaboration with clinical care teams for weight loss and is marketed as a specialist dietary management app and as such does not mirror a typical smartphone app such as MyFitnessPal. |
| Badau, D., & Badau, A. (2018). Identifying the incidence of exercise dependence attitudes, levels of body perception, and preferences for use of fitness technology monitoring. *International Journal of Environmental Research and Public Health*, *15*(12), 2614. <https://doi.org/10.3390/ijerph15122614> | No response from authors. | The authors did measure FDT use and excessive exercise through the Exercise Dependence Scale-R and the Compulsive Exercise Test, but did not conduct or report on any analysis between these two variables. The authors were contact to ask for this association, but there was no response. |
| Berry RA. 2020 Longitudinal relationships between fitness tracking and disordered eating in undergraduate women. *Doctoral Dissertation, Northeastern University, USA,* <http://hdl.handle.net/2047/D20397590> | Unpublished Duplicate | Is a duplicate of the Berry et al. (2024) paper which has been included as it has been subject to a peer review process. |
| Bohrer, B. K., Chen, Y., Christensen, K. A., Forbush, K. T., Thomeczek, M. L., Richson, B. N., Chapa, D. A. N., Jarmolowicz, D. P., Gould, S. R., Negi, S., Perko, V. L., & Morgan, R. W. (2023). A pilot multiple-baseline study of a mobile cognitive behavioral therapy for the treatment of eating disorders in university students. *The International journal of eating disorders*, *56*(8), 1623–1636. <https://doi.org/10.1002/eat.23987> | Wrong study design. | App offered CBT guided self-help and as such does not mirror a typical smartphone app such as MyFitnessPal. |
| Bulik, C. M., Butner, J. E., Tregarthen, J., Thornton, L. M., Flatt, R. E., Smith, T., Carroll, I. M., Baucom, B. R. W., & Deboeck, P. R. (2020). The binge eating genetics initiative (BEGIN): study protocol. *BMC psychiatry*, *20*(1), 307. <https://doi.org/10.1186/s12888-020-02698-7> | Wrong study design. | This project utilised both Apple Watches and an app called Recovery Record. The watches, however, were used to record step number and as such not used in the context of self-monitoring. Additionally, Recovery Record is an app designed to help individuals recovering from the ED, and as such it was decided that it was outwith the scope of the current review which instead focused on health and fitness orientated FDTs. |
| Carels, R. A., Hlavka, R., Selensky, J. C., Solar, C., Rossi, J., & Caroline Miller, J. (2019). A daily diary study of internalised weight bias and its psychological, eating and exercise correlates. *Psychology & Health, 34*(3), 306–320. <https://doi.org/10.1080/08870446.2018.1525491> | Wearable not used for self-monitoring purposes. | FDTs were provided to the participants, however, they were used to record exercise not for personal use or for self-monitoring. |
| Dakin, C. A., Finlayson, G., Horgan, G., Palmeira, A. L., Heitmann, B. L., Larsen, S. C., Sniehotta, F. F., & Stubbs, R. J. (2023). Exploratory analysis of reflective, reactive, and homeostatic eating behaviour traits on weight change during the 18-month NoHoW weight maintenance trial. *Appetite*, *189*, 106980. <https://doi.org/10.1016/j.appet.2023.106980> | Wrong study design. | Although participants reported experiencing disordered eating behaviours (binge eating). This app is designed to be used as part of a toolkit and as such does not mirror a typical smartphone app such as MyFitnessPal. |
| Durrer, D., Kowatsch, T., L'Allemand-Jander, D., Büchter, D., Pletikosa Cvijikj, L., Maass, W. & Schutz, Y. (2015). Ambulatory health information system for obesity prevention and treatment (pathmate) tailored for teenagers: a preliminary longitudinal study. *Obes Facts*, *8*(1). | No measure of wearable use. | The paper did not mention using a measure to assess wearable use. Additionally, they created a tailored interactive intervention for weight loss (an online weight loss intervention), and FDT was used to measure physical activity, not for self-monitoring. |
| Edwards A. (2017). The impact of body image on Fitbit use: a comparison across genders. *Health Information and Libraries Journal*, *34*(3), 247–251. <https://doi.org/10.1111/hir.12188> | Qualitative | This study conducted interviews and as such as excluded as there was no quantitative data. |
| Eikey, E.V., Chen, Y., Zheng, K. (2019). Do recovery apps even exist?: Why college women with eating disorders use (but not recommend) diet and fitness apps over recovery apps. In: Taylor, N., Christian-Lamb, C., Martin, M., Nardi, B. (eds) *Information in Contemporary Society.* iConference 2019. *Lecture Notes in Computer Science*(11420), Springer, Cham. <https://doi.org/10.1007/978-3-030-15742-5_69> | Qualitative |  |
| Elling, C.E., Geiger, A.M., Goodman, W. & Wolf, J.M. (2016) Despite general increases in body-esteem with age, low weight satisfaction is linked to increased physical activity in middle-aged and older adults, *Psychoneuroendocrinology*, *71*(38). <https://doi.org/10.1016/j.psyneuen.2016.07.101> | Wearable not used for self-monitoring purposes. | Participants were provided with Fitbits; however, they were not required to use them and they acted as a pedometer to measure participant step count.  Additionally, there was no measure of disordered eating but instead body-esteem. |
| Ferrin, S.N., Rouse, M., Shan, M. & Aalsma, M.C. (2020). Social media, fitness, and nutrition application usage among adolescents and adults with eating disorders. *Journal of Adolescent Health*. *66*(2). <https://doi.org/10.1016/j.jadohealth.2019.11.125> | No measure of disordered eating | Although this study looked at wearable use in people with an eating disorder, only the presence or absence of use was reported, e.g., that X number of participants used X device. The authors were contacted to query whether any measure such as the EDDS had been used, or if any further analysis had been done but there had not. |
| Flatt, R. E., Thornton, L. M., Smith, T., Mitchell, H., Argue, S., Baucom, B. R. W., Deboeck, P. R., Adamo, C., Kilshaw, R. E., Shi, Q., Tregarthen, J., Butner, J. E., & Bulik, C. M. (2022). Retention, engagement, and binge-eating outcomes: Evaluating feasibility of the binge-eating genetics initiative study. *The International Journal of Eating Disorders*, *55*(8), 1031–1041. <https://doi.org/10.1002/eat.23726> | Wrong study design. | Recovery Record is an app designed to help individuals recovering from the ED. As such, it was decided that it was out with the scope of the current review that instead focuses on health and fitness orientated FDTs. Additionally, this paper focused on evaluating the app and its functions itself. |
| Goodyear, V.A. & Armour, K.M. (2018) Young people’s perspectives on and experiences of health-related social media, apps, and wearable health devices. *Social Sciences*. *7*(8)  <https://doi.org/10.3390/socsci7080137> | Review | This paper was a review and synthesis of three separate studies examining young people perspectives on social media and FDTs. The majority of work conducted was qualitative and in the quantitative study (Goodyear et al., 2018c), there is no mention of using an eating disorder measure. |
| Hahn, S. (2020). *Examining the Relationships Between Weight-Related Self-Monitoring and Eating Disorder Risk Among College Students* [Doctoral thesis, University of Michigan]. | Duplicate |  |
| Hawley, A. M. (2022). *Eating Disorder Risk and Calorie Tracking Application Usage Among University Students*[Master's thesis, Kent State University]. | No response from authors | The authors did use an eating disorder measure (Eating Disorder Diagnostic Scale) and assessed whether or not participants used a FDT, however, the EDDS was not interpreted. The author was contacted to ask for the results, but there was no response. |
| Honary, M., Bell, B. T., Clinch, S., Wild, S. E., & McNaney, R. (2019). Understanding the role of healthy eating and fitness mobile apps in the formation of maladaptive eating and exercise behaviors in young people. *JMIR mHealth and uHealth*, *7*(6), e14239. <https://doi.org/10.2196/14239> | Qualitative | This paper used qualitative methods including workshops and a review of the top 100 healthy eating and fitness apps. As such it was excluded as only quantitative literature was included in the review. |
| Kilshaw, R. E., Adamo, C., Butner, J. E., Deboeck, P. R., Shi, Q., Bulik, C. M., Flatt, R. E., Thornton, L. M., Argue, S., Tregarthen, J., & Baucom, B. R. W. (2022). Passive sensor data for characterizing states of increased risk for eating disorder behaviors in the digital phenotyping arm of the binge eating genetics initiative: protocol for an observational study. *JMIR research protocols*, *11*(6), e38294. <https://doi.org/10.2196/38294> | Wrong study design | This project utilised both Apple Watches and an app called Recovery Record. The watches, however, were used to record step number and as such not used in the context of self-monitoring. Additionally, Recovery Record is an app designed to help individuals recovering from the ED, and as such it was decided that it was out with the scope of the current review which instead focused on health and fitness orientated FDTs. |
| Li, X., Petersen, C.L., Roderka, M.N., Gooding, T.L. & Batsis, J.A. (2020). Physical activity is associated with positive feelings with a weight management program. *Journal of the American Geriatrics Society.* *(68)*. | No measure of disordered eating. |  |
| McClure, S. M. (2013). *"It's Just Gym": Physicality and Identity among African American Adolescent Girls* [Doctoral dissertation, Case Western Reserve University]. | Wearable not used for self-monitoring purposes. | FDTs were provided to the participants, however, they were used to record exercise not for personal use or for self-monitoring. |
| Pech, M., Correll, C. U., Schmidt, J., Zeeck, A., Hofmann, T., Busjahn, A., & Haas, V. (2024). The Relationship between Patient Self-Reported, Pre-Morbid Physical Activity and Clinical Outcomes of Inpatient Treatment in Youth with Anorexia Nervosa: A Pilot Study. *Nutrients*, *16*(12), 1889. <https://doi.org/10.3390/nu16121889> | Wearable not used for self-monitoring purposes. |  |
| Presseller, E. K., Lampe, E. W., Zhang, F., Gable, P. A., Guetterman, T. C., Forman, E. M., & Juarascio, A. S. (2023). Using Wearable Passive Sensing to Predict Binge Eating in Response to Negative Affect Among Individuals With Transdiagnostic Binge Eating: Protocol for an Observational Study. *JMIR research protocols*, *12*, e47098. <https://doi.org/10.2196/47098> | Wearable not used for self-monitoring purposes. |  |
| Rethorst, C. D., Githinji, P., Seguin-Fowler, R. A., MacMillan Uribe, A. L., Szeszulski, J., & Liao, Y. (2023). Real-time Assessment of the Bidirectional Relationship Between Affective States and Glucose: Protocol for a 14-Day Observational Study. *JMIR research protocols*, *12*, e45104. <https://doi.org/10.2196/45104> | Wearable not used for self-monitoring purposes. |  |
| Riverso, A. (2015). *The potential for Greek housing to shape diet and exercise patterns: An exploratory intra-Greek comparison* (Doctoral dissertation, Syracuse University). | No measure of wearable use. | Participants created a food record on MyfitnessPal for the purposes of informing the researchers of their eating habits and caloric intake. There was no measure of whether participants used this over the week or simply created a record for the purpose of the study. |
| Schmidt, M. E., Hiensch, A., Depenbusch, J., Clauss, D., Monninkhof, E., Pelaez, M., ... & Steindorf, K. (2024). 269MO The effects of exercise on sexual health and breast cancer-specific symptom burden in women with metastatic breast cancer: Results of the multinational randomized PREFERABLE-EFFECT trial. *ESMO Open*, *9*. | No measure of disordered eating. |  |
| Wade, G. Marshall, M., Woodford, J., Simms, C., Robson, D., Geddes, L. & Lange, C. (2024) The Smarter Way to a Fitter You. *NewScientist, 263(3501) 32.* <https://doi.org/10.1016/S0262-4079(24)01362-9> | Qualitative |  |
| Weiner, L. S., Nagel, S., Irene Su, H., Hurst, S., Levy, S. S., Arredondo, E. M., Hekler, E., & Hartman, S. J. (2023). A remotely delivered, peer-led intervention to improve physical activity and quality of life in younger breast cancer survivors. *Journal of Behavioral Medicine*, *46*(4), 578–593. <https://doi.org/10.1007/s10865-022-00381-8> | No measure of disordered eating. | This study did not use a measure of disordered eating, additionally the data collected pertaining to possible beneficial and adverse outcomes of FDTs was qualitative. |
| Weiner, L. S., Nagel, S., Su, H. I., Hurst, S., Levy, S. S., Arredondo, E. M., Hekler, E., & Hartman, S. J. (2023). Correction to: A remotely delivered, peer-led intervention to improve physical activity and quality of life in younger breast cancer survivors. *Journal of behavioral medicine*, *46*(4), 707–708. <https://doi.org/10.1007/s10865-022-00390-7> | Duplicate | Duplicate of previously excluded paper reporting on a correction. |
| Wons, O., Lampe, E., Patarinski, A. G., Schaumberg, K., & Juarascio, A. (2023). Change in adaptive and maladaptive exercise and objective physical activity throughout CBT for individuals with eating disorders. *Eating and weight disorders : EWD*, *28*(1), 40. <https://doi.org/10.1007/s40519-023-01566-z> | Wearable not used for self-monitoring purposes. |  |
| Wons, O., Lampe, E., Patarinski, A.G., Schaumberg, K., Butryn, M., & Juarascio, A. (2022). Perceived influence of wearable fitness trackers on eating disorder symptoms in a clinical transdiagnostic binge eating and restrictive eating sample. *Eating and weight disorders*, *27*(8), 3367–3377. <https://doi.org/10.1007/s40519-022-01466-8> | Wearable not used for self-monitoring purposes. | Although this study both included a measure of disordered eating and FDT use, participants were supplied FDTs to track their behaviour but asked not to use the devices for self-monitoring. |
| Wons, O.B. (2021*) Characterization and Assessment of Change in Adaptive and Maladaptive Exercise, and Total Objectively Measured Physical Activity Over a 12-Week CBT Treatment for Individuals with Transdiagnostic Binge Eating and Restrictive* Eating. [Master’s Thesis, Drexel University]. | Duplicate |  |
| Wons, O.B., Michael, M.L., Lin, M., & Juarascio, A.S. (2021). Characterizing rates of physical activity in individuals with binge eating disorder using wearable sensor technologies and clinical interviews. *European Eating Disorders Review: the Journal of the Eating Disorders Association*, *29*(2), 292–299. <https://doi.org/10.1002/erv.2811> | Wearable not used for self-monitoring purposes. | Although this study both included a measure of disordered eating and FDT use, participants were supplied FDTs to track their behaviour but asked not to use the devices for self-monitoring. |
| Zopf, E. Demonstrating the (cost-)effectiveness of a personalized live-remote exercise intervention for cancer survivors using a super umbrella randomized controlled trial: the LION-RCT. *Cabrini Institute.* | Protocol |  |
